# Immunohistochemical analysis of Bcl-2 protein in early squamous cell carcinoma of the bronchus treated with photodynamic therapy

**DOI:** 10.1054/bjoc.1999.0936

**Published:** 2000-01-18

**Authors:** T Kawaguchi, S Yamamoto, N Naka, K Okishio, S Atagi, M Ogawara, S Hosoe, M Kawahara, K Furuse

**Affiliations:** Department of Internal Medicine and Pathology, National Kinki Central Hospital for Chest Diseases, 1180 Nagasone-cho, Sakai, Osaka, 591, Japan

**Keywords:** Bcl-2 protein, early squamous cell carcinoma, bronchus, photodynamic therapy

## Abstract

Photodynamic therapy (PDT) in early squamous cell carcinoma of the bronchus has been shown to result in complete response (CR) and cure. However, local recurrence after PDT develops frequently even after complete remission. Because the effect of PDT had been reported to depend on apoptosis, and apoptosis is inhibited by bcl-2 protein, the relationship between the expression of bcl-2 protein and local recurrence after PDT was examined immunohistochemically. From 1983 to 1997, 50 patients with 59 early squamous cell carcinoma of the bronchus received PDT, and a CR was obtained in 43 lesions (72.8%). As there was no recurrence among tumours that were disease-free for more than 2 years, in this study the tumours were defined as cured when recurrence did not occur 2 years subsequent to the receiving of PDT. Of these CR lesions, 31 carcinomas (53.4%) resulted in a cure. Bcl-2 immunoreactivity was detected in 23 tumours (46.9%) and p53 immunoreactivity was detected in 22 tumours (44.9%). When all tumours were divided into either a large tumour with a longitudinal tumour length of 10 mm or more, or a small tumour with a length of less than 10 mm, the large tumour expressed more bcl-2 protein than the small tumour (*P* = 0.0155). The degree of bcl-2 expression was significantly related with tumour size (*P* = 0.0155). The expression of bcl-2 and p53 protein was not associated with the cure rate due to PDT. Tumour length and T status in TNM staging were significantly related to the cure by univariate analysis. T status was the only predictor of the cure according to mutivariate analysis. Of 42 CR lesions, the expression of neither bcl-2 nor p53 protein was associated with local recurrence; only T status was significantly associated (*P* = 0.008). The relationship between the expression of oncoprotein and local recurrence after PDT was not documented in this study. The success of PDT may depend on the exact assessment of tumour size under optimized PDT illumination. © 2000 Cancer Research Campaign


*British Journal of Cancer* (2000) **82**(2), 418–423
© 2000 Cancer Research Campaign
Article no. bjoc.1999.0936

### Immunohistochemical analysis of Bcl-2 protein in early squamous cell carcinoma of the bronchus treated with photodynamic therapy

**T Kawaguchi, S Yamamoto, N Naka, K Okishio, S Atagi, M Ogawara, S Hosoe, M Kawahara and K Furuse**

Department of Internal Medicine and Pathology, National Kinki Central Hospital for Chest Diseases, 1180 Nagasone-cho, Sakai, Osaka, 591 Japan

**Summary** Photodynamic therapy (PDT) in early squamous cell carcinoma of the bronchus has been shown to result in complete response (CR) and cure. However, local recurrence after PDT develops frequently even after complete remission. Because the effect of PDT had been reported to depend on apoptosis, and apoptosis is inhibited by bcl-2 protein, the relationship between the expression of bcl-2 protein and local recurrence after PDT was examined immunohistochemically. From 1983 to 1997, 50 patients with 59 early squamous cell carcinoma of the bronchus received PDT, and a CR was obtained in 43 lesions (72.8%). As there was no recurrence among tumours that were disease-free for more than 2 years, in this study the tumours were defined as cured when recurrence did not occur 2 years subsequent to the receiving of PDT. Of these CR lesions, 31 carcinomas (53.4%) resulted in a cure. Bcl-2 immunoreactivity was detected in 23 tumours (46.9%) and p53 immunoreactivity was detected in 22 tumours (44.9%). When all tumours were divided into either a large tumour with a longitudinal tumour length of 10 mm or more, or a small tumour with a length of less than 10 mm, the large tumour expressed more bcl-2 protein than the small tumour ($P = 0.0155$). The degree of bcl-2 expression was significantly related with tumour size ($P = 0.0155$). The expression of bcl-2 and p53 protein was not associated with the cure rate due to PDT. Tumour length and T status in TNM staging were significantly related to the cure by univariate analysis. T status was the only predictor of the cure according to mutivariate analysis. Of 42 CR lesions, the expression of neither bcl-2 nor p53 protein was associated with local recurrence; only T status was significantly associated ($P = 0.008$). The relationship between the expression of oncoprotein and local recurrence after PDT was not documented in this study. The success of PDT may depend on the exact assessment of tumour size under optimized PDT illumination. © 2000 Cancer Research Campaign

**Keywords**: Bcl-2 protein; early squamous cell carcinoma; bronchus; photodynamic therapy

Currently the most suitable treatment for early squamous cell carcinoma of the bronchus is photodynamic therapy (PDT). PDT in early squamous carcinoma has demonstrated complete response (CR) and cure (Furuse et al, 1993). However, many reports have shown an almost 20% local recurrence after PDT (Edell et al, 1992; Furuse et al, 1993; Sutedja et al, 1994), and so it is important to determine the factors which influence the onset of local reccurence.

One of the factors could be inherent tumour cell resistance. Pass (1993) showed that if there is regrowth of tumour after PDT, it usually occurs at the edge of the tumour and the regrowth could be due to inherent tumour cell resistance, hypoxia, inadequate drug uptake, or inadequate drug and/or light dose concentration.

The effect of PDT is thought to be non-specific damage due to the production of single oxygen. Recent studies, though, indicate that PDT can induce apoptosis both in vivo and in vitro. Agarwal et al (1991, 1993) showed that PDT stimulates phospholipases on cell membranes, and initiates apoptosis in mouse lymphoma cells. In addition, they demonstrated that PDT can cause increased synthesis of ceramide, and that ceramide production is linked to apoptosis in PDT treated L5178Y-R cells (Separovic et al, 1997). Though PDT photo-oxidizes cellular targets, the control systems and mechanisms remain largely unknown. Fisher et al (1997) reported that photosensitivity was increased in HL-60 cells expressing wild-type p53, and that photosensitizer-mediated oxidative stress can induce apoptosis through a p53-independent mechanism. The bcl-2-expressing cell was also reported to be relatively resistant to PDT in Chinese ovary cell (He et al, 1996).

Bcl-2, a proto-oncogene which is expressed in a variety of malignant tumours, works as an inhibitor of apoptosis. The p53 is a tumour suppressor gene, and is the most commonly mutated gene occurring in cancer. Because the bcl-2 expression is negatively regulated by the wild-type p53, the *p53* gene mutations inhibit apoptosis as well as cause a loss of tumour suppressor function. The mutated p53 is reported to be detected by immunohistochemical staining (Nishio et al, 1996).

In this paper, the clinical outcome of patients in early stage bronchogenic carcinoma treated with PDT was studied. The bcl-2 and p53 protein in bronchoscopic specimens before treatment were examined immunohistochemically. Whether expression of bcl-2 and p53 protein can be predictive of local recurrence after PDT was assessed.

*Received 12 January 1999*
*Revised 20 July 1999*
*Accepted 3 August 1999*

*Correspondence to:* T Kawaguchi

### MATERIALS AND METHODS

### Patients and therapy

From 1983 to 1997, 50 patients with 59 early squamous cell carcinoma of the bronchus received PDT in this institute. Patient characteristics are shown in Table 1. The age of the patients ranged

**Table 1** Patient characteristics of 50 patients with 59 carcinomas

| | |
|---|---|
| Age | |
| Median | 68 |
| Range | 48–80 |
| Sex (male/female) | 46/4 |
| Stage (no. of carcinomas) | |
| TisN0M0 | 9 |
| T1n0m0 | 50 |
| Location of tumour | |
| Trachea | 6 |
| Main bronchus | 0 |
| Lobar bronchus | 16 |
| Segmental bronchus | 32 |
| Subsegmental bronchus | 5 |
| Tumour morphology | |
| Superficial type | 34 |
| Nodular type | 15 |
| Polypoid type | 10 |
| Estimated length of tumour (mm) | |
| >20 | 4 |
| 20– >10 | 11 |
| ≤10 | 44 |
| Distal tumour margin | |
| Clearly visible | 45 |
| Not clearly visible | 14 |

from 48 to 80 years old (median 68 years); 46 were male and four were female. According to the TNM classification, nine cases were TisN0M0 and 50 cases T1N0M0 (Mountain, 1986). The length of longitudinal tumour extent on the bronchial surface was estimated by a bronchoscopist. This measurement was estimated using open-biopsy forceps of known diameter or a special bronchial scale, and photographs were taken at the same time. Twenty-eight cases underwent PDT by excimer dye laser, 25 cases were treated using argon dye laser and six underwent PDT with YAG OPO laser.

PDT was performed using a flexible fibrebronchoscope, through which a laser beam was delivered to the lesions. All procedures were performed under local anaesthesia. All patients received 3 mg $kg^{-1}$ photofrin I or 2 mg $kg^{-1}$ photofrin II intravenously, 48 h before PDT. Treatment energies ranged from 100 to 300 J $cm^{-2}$ (median 200 J $cm^{-2}$) in the excimer dye laser and YAG-OPO laser, and from 30 to 810 J $cm^{-2}$ (median 290 J $cm^{-2}$) in the argon dye.

Of the tumours treated with PDT, 43 lesions (72.8%) obtained a CR. A CR was defined as no demonstrable tumour by biopsy and/or brushing, and no tumour visible on chest roentgenogram for at least 4 weeks.

Extended, long follow-up results of the lesions treated with PDT have been studied. Median follow-up time after PDT was 7 years and 2 months with a range of 3 months to 14 years. One CR tumour was lost in follow-up. Of 42 CR tumours, 11 lesions recurred locally within 2 years follow-up. There was no recurrence among tumours which were disease-free for more than 2 years. Therefore, in this study, the tumour was defined to be cured if there was no recurrence after 2 years subsequent to PDT. As a result, 31 lesions (53.4%) obtained a cure after a single course of PDT.

### Tumour biopsies

Forty-nine biopsy specimens were collected from 59 tumours. Ten lesions were excluded because they were so small as to be unsuitable for immunohistochemical examination. These specimens were fixed in 10% formaldehyde solution and embedded in paraffin. Four-micrometre-thick sections were cut and mounted on glass slides.

### Immunohistochemical staining

Immunohistochemical studies were performed by the streptavidin–biotin method. Sections were deparaffinized in xylene and absolute alcohol and autoclaved at 121°C with distilled water for 20 min. After cooling 10% rabbit serum was plased on the slides to reduce background staining. For bcl-2 immunostaining, specimens were incubated overnight with a 1:20 dilution of an anti-bcl-2 monoclonal antibody (Dako A/S, Glostrup, Denmark) at room temperature. For p53 immunostaining specimens were incubated for an hour with a 1:50 dilution of an anti-p53 monoclonal antibody, DO-7 (Dako A/S, Glostrup, Denmark) at room temperature. The sections were then incubated with biotinylated donkey anti-mouse immunoglobulin G for 30 min. The slides were treated with streptavidin–peroxidase for 30 min, reacted in phosphate-buffered saline (PBS) containing diaminobenzidine, counterstained with haematoxylin and mounted.

A tumour was considered to be immunopositive for bcl-2 when the number of cells with stained cytoplasm exceeded 5%, and immunopositive for p53 when the number of cells with stained nuclei exceeded 10%. According to Nishio (1996), a 10% cutoff value reliably indicates the presence of mutated p53.

Slides were interpreted by two investigators (TK and SY) who reached agreement without prior knowledge of the clinical outcome.

### Statistical analysis

The relationship between the expression of bcl-2, p53 protein and clinical data was examined by univariate analysis. Pretreatment variables, including expression of bcl-2 and p53 protein, were investigated for any possible relationship to cure and local recurrence after PDT, first by univariate analysis, and subsequently by application of a multiple regression model. The univariate analysis was based on $2 \times 2$ tables, and differences were tested by use of the $\chi^2$ test or Fisher's exact test.

### RESULTS

The bcl-2 immunoreactivity was localized in the cytoplasm of neoplastic cells and bcl-2 immunopositivity was detected in 23 tumours (46.9%); p53 immunoreactivity was confined to the nuclei of neoplastic cells and p53 immunopositivity was detected in 22 tumours (44.9%) (Figures 1 and 2).

The relationship between the expression of bcl-2, p53 protein and clinical data is shown in Table 2. When all the tumours were divided by median value of age (> 68 vs 68≥), T status in TNM staging (Tis vs T1) and tumour morphology (superficial type vs non-superficial type), there was no significant relationship between the expression of bcl-2, p53 protein and these variables. When the tumours were divided into a large tumour group with a longitudinal tumour length of 10 mm or more, and a small tumour group with a length of less than 10 mm, bcl-2 expression was found in nine of 14 (64.3%) tumours in the large and eight of 33 (24.2%) tumours in the small tumour group. This difference was statistically significant ($P = 0.0155$).

*© 2000 Cancer Research Campaign*

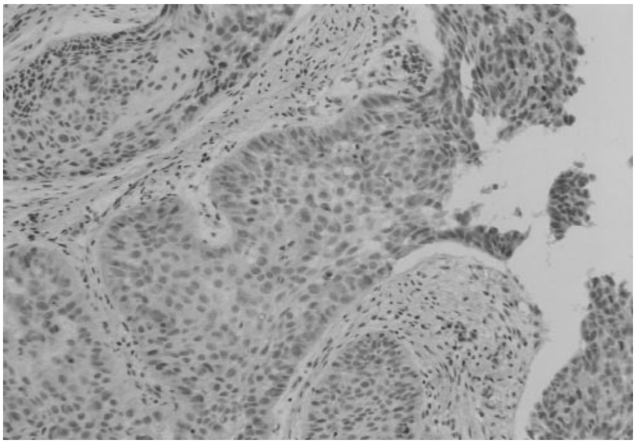

**Figure 1** Immunohistochemical staining of bcl-2 protein in early squamous cell carcinoma of the bronchus. The immunoreactivity was localized in the cytoplasm of neoplastic cells

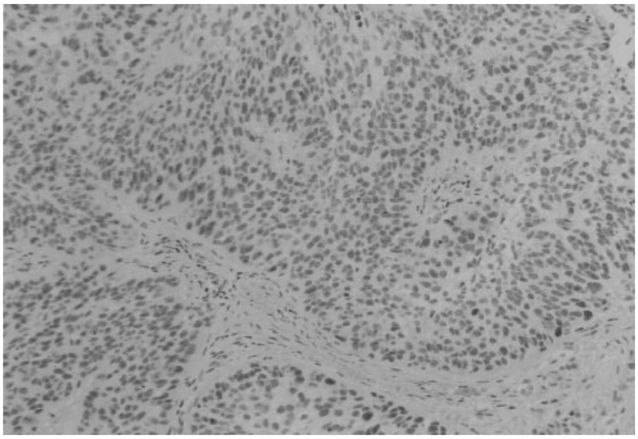

**Figure 2** Immunohistochemical staining of p53 protein in early squamous cell carcinoma of the bronchus. The immunoreactivity was confined to the nuclei of neoplastic cells

**Table 2** Relationship between expression of bcl-2, p53 and clinical data

| Variable | bcl-2 | | | p53 | | |
|---|---|---|---|---|---|---|
| | Negative | Positive | *P* | Negative | Positive | *P* |
| Age | | | | | | |
| >68 | 15 | 9 | 0.9248 | 13 | 11 | 0.9897 |
| ≥68 | 15 | 7 | | 11 | 11 | |
| T status in TNM stage | | | | | | |
| Tis | 6 | 2 | 0.8229 | 4 | 4 | 0.8433 |
| T1 | 26 | 15 | | 22 | 19 | |
| Tumour morphology | | | | | | |
| Superficial type | 20 | 9 | 0.5746 | 17 | 12 | 0.2813 |
| Non-superficial type | 12 | 8 | | 9 | 11 | |
| Length of tumour | | | | | | |
| >10 | 5 | 9 | 0.0155 | 10 | 5 | 0.3382 |
| ≥10 | 25 | 8 | | 16 | 18 | |

The follow-up outcome of PDT and clinicopathological data were examined. As mentioned above, the 31 carcinomas were defined as cured. The relationship between the cure due to PDT and pretreatment variables, including expression of bcl-2 and p53 protein, are shown in Table 3. Expression of bcl-2 and p53 protein was not associated with the cure after PDT. Visibility of the distal tumour margin, tumour location and tumour morphology also showed no association with the cure. Both the tumour length and T status in TNM staging were significantly related to the cure by univariate analysis. The factors influencing the cure due to PDT were examined by multivariate analysis. As shown in Table 4, only T status in TNM staging was significantly related to the cure.

Moreover, the factors considered to influence the local recurrence after PDT were evaluated. Because one CR tumour was lost in follow-up, 42 CR tumours were examined. As described above, 11 lesions recurred locally. The results of the relationship between local recurrence and clinicopathological data are shown in Table 5. T status in TNM staging was significantly associated with the local recurrence according to univariate analysis ($P$ = 0.0320); expression of bcl-2 and p53 protein was not associated with local recurrence. The results of the multivariate analysis of the same 42 CR lesions are shown in Table 6. T status in TNM staging was also significantly associated with local reccurence after PDT

**Table 3** Relationship between the cure and clinicopathological data; Results of univariate analysis

| | Cure | | |
|---|---|---|---|
| | (–) | (+) | *P* |
| bcl-2 | | | |
| (–) | 15 | 17 | 0.3735 |
| (+) | 11 | 6 | |
| p53 | | | |
| (–) | 16 | 10 | 0.4994 |
| (+) | 11 | 12 | |
| Length of tumour | | | |
| >10 | 12 | 3 | 0.0066 |
| 10≥ | 15 | 28 | |
| T status in TNM stage | | | |
| Tis | 0 | 9 | 0.0073 |
| T1 | 27 | 22 | |
| Tumour margin | | | |
| Clear | 17 | 27 | 0.0665 |
| Not clear | 10 | 4 | |
| Tumour location | | | |
| Distal to segmental bronchus | 19 | 17 | 0.2239 |
| Proximal to lobar bronchus | 8 | 14 | |
| Tumour morphology | | | |
| Superficial type | 17 | 15 | 0.3334 |
| Non-superficial type | 10 | 16 | |

© 2000 Cancer Research Campaign

**Table 4** Factors influencing the cure due to PDT; results of logistic regression analysis

| Variable | β | s.e.m. | P |
|---|---|---|---|
| bcl-2 (–) vs (+) | –0.060 | 0.165 | 0.707 |
| p53 (–) vs (+) | 0.080 | 0.149 | 0.594 |
| Length of tumour (> 10 vs 10≥) | –0.152 | 0.190 | 0.393 |
| T status in TNM stage (Tis vs T1) | –0.361 | 0.190 | 0.0167 |
| Tumour margin (Clear vs not clear) | –0.044 | 0.176 | 0.784 |
| Tumour location (Distal to segmental bronchus vs proximal to lobar bronchus) | –0.178 | 0.149 | 0.218 |
| Tumour morphology (Superficial type vs non-superficial type) | 0.146 | 0.148 | 0.332 |

**Table 5** Relationship between local recurrence and clinicopathological data in 42 CR lesions; Results of univariate analysis

| | Local recurrence | | |
|---|---|---|---|
| | (–) | (+) | *P* |
| bcl-2 | | | |
| (–) | 17 | 8 | 0.9556 |
| (+) | 6 | 4 | |
| p53 | | | |
| (–) | 10 | 6 | 0.9157 |
| (+) | 12 | 6 | |
| Length of tumour | | | |
| >10 | 3 | 1 | 0.6777 |
| 10≥ | 27 | 11 | |
| T status in TNM stage | | | |
| Tis | 9 | 0 | 0.0320 |
| T1 | 21 | 12 | |
| Tumour margin | | | |
| Clear | 26 | 10 | 0.7803 |
| Not clear | 4 | 2 | |
| Tumour location | | | |
| Distal to segmental bronchus | 16 | 10 | 0.0705 |
| Proxymal to lobar bronchus | 14 | 2 | |
| Tumour morphology | | | |
| Superficial type | 14 | 9 | 0.1166 |
| Non-superficial type | 15 | 3 | |

($P = 0.008$). The products of bcl-2 and p53, tumour length, distal tumour margin, tumour location and tumour morphology were not associated with the local recurrence after PDT.

### DISCUSSION

PDT is an effective treatment modality in a variety of solid cancers due to the lack of cumulative toxicity and slight mucosal damage (Kawaguchi et al, 1998). PDT in early-stage bronchogenic carcinoma has been shown to result in 74–85% CR. An important factor in regulating CR was longitudinal tumour extent. On the other hand, tumour location, tumour morphology and distal tumour margin were not important factors. Indeed, an excellent 97.8% CR rate could be obtained with limited longitudinal extent (≤ 1 cm) (Furuse et al, 1993). With regard to long-term follow-up in patients with centrally located early-stage bronchogenic carcinoma, complete cure cases treated with PDT were reported (Kato et al, 1986). Cortese (1997) showed that nine of 21 patients (43%) with early-stage squamous cell carcinoma who were candidates for PDT could be spared surgical resection, and the mean duration of follow-up for these nine patients was 68 months. In this study there was no recurrence among patients who were disease-free for more than 2 years. The 31 patients (53.4%) were considered to be cured if there was no recurrence 2 years post-PDT. Therefore, PDT could be considered an effective method to treat and cure an early-stage bronchogenic carcinoma. However, it is still problematic that 11 local recurrences in 42 CR lesions have occurred within 2 years after PDT in our study. Several reports also showed that there was almost 20% local recurrence rate after PDT (Edell et al, 1992; Furuse et al, 1993; Sutedja et al, 1994). To establish the treatment of PDT for early-stage bronchogenic carcinoma, it is important to determine the factors that influence the onset of local recurrence.

**Table 6** Factors influencing the local recurrence after PDT (42 CR lesions); results of logistic regression analysis

| Variable | β | s.e.m. | *P* |
|---|---|---|---|
| bcl-2 (–) vs (+) | –0.102 | 0.184 | 0.557 |
| p53 (–) vs (+) | 0.073 | 0.182 | 0.703 |
| Length of tumour (>10 vs 10≥) | 0.144 | 0.351 | 0.550 |
| T status in TNM stage (Tis vs T1) | –0.481 | 0.189 | 0.008 |
| Tumour margin (Clear vs not clear) | –0.081 | 0.245 | 0.684 |
| Tumour location (Distal to segmental bronchus vs proximal to lobar bronchus) | –0.273 | 0.176 | 0.127 |
| Tumour morphology (Superficial type vs non-superficial type) | 0.172 | 0.179 | 0.353 |

There are many possible reasons for local recurrence after PDT. In this study, we focused on the inherent tumour cell resistance to PDT, because recent studies showed that the anti-tumour effect of PDT has depended on apoptosis (Agarwal et al, 1991, 1993). Oncoproteins such as bcl-2 or p53, which regulate apoptosis, have also been documented to influence the outcome of PDT in vitro (He et al, 1996; Fisher et al, 1997). Although there are many clinical reports on the impact of bcl-2 or p53 protein expression on the response of chemotherapy and radiotherapy, little clinical data have been available concerning the relationships between oncoprotein and PDT. Therefore, using immunohistochemical techniques, we retrospectively studied the effect of oncoprotein on the cure rate and local recurrence after PDT.

Bcl-2 immunoreactivity was detected in 23 tumours (46.9%) and p53 immunoreactivity detected in 22 tumours (44.9%). When the tumour length was divided into two groups, 10 mm or more or less than 10 mm in length, the larger tumours expressed the bcl-2 protein more significantly ($P = 0.0155$); the degree of relationship of bcl-2 expression to tumour size was significant ($P = 0.0155$). Expression of bcl-2 protein can be observed at a very early stage in the development of lung cancer. Walker (1995) showed that aberrant bcl-2 expression was identified in mild dysplasia using immunohistochemical analysis, and that it correlated with an increasing grade of dysplasia. Koukourakis et al (1997) showed that a strong inverse relationship was found between the bcl-2 expression and tumor angiogenesis. They suggested that bcl-2

© 2000 Cancer Research Campaign positive tumours are present at an early stage of progression, with loss of bcl-2 expression a relatively late event in the pathogenesis of the lung cancer. Early-stage bronchogenic carcinoma in this study, which has little or no potential to metastasize, may be evident at an early stage of progression. Therefore, the degree of bcl-2 expression, as well as the degree of bronchial dysplasia, might be significantly related to tumour size ($P = 0.0155$).

In our study, the expression of bcl-2 and p53 protein was not documented as a poor prognostic indicator of PDT. Tumour volume, estimated by T status in TNM staging and tumour length, was the significant indicator of the cure according to univariate analysis. By mutivariate analysis, only T status in TNM staging was the single significant indicator of the cure. In addition, with regard to the local recurrence after PDT, only T status in TNM staging was also significantly associated with it. Since the expression of bcl-2 and p53 protein did not influence the prognosis of PDT, this treatment may not be associated with apoptosis in the clinical setting as seen in vitro. Indeed, He et al (1994) described that apoptosis induced by PDT in vitro depends on the kind of photosensitizer and cell line. Luo (1997) showed that the mode of cell death can be shifted from an apoptotic to a necrotic response as a function of the PDT dose. Higher light doses yielded progressively more membrane photodamage and inhibited the apoptotic response. In a clinical setting, it is possible that higher light doses and higher photosensitizer concentration are achieved in some cases, because subepitheial fibrosis was found in histological examination of clinical PDT (Kawaguchi et al, 1998). In addition, the tumour vessel is considered to be an important target of PDT (Star et al, 1986). Although vascular damage can also lead to apoptosis, these relationships need further investigation.

Our study showed that the success of PDT is related to the tumour volume, especially T status in TNM staging, and the clinical T status could reflect tumour depth. Although bronchoscopic techniques have been well developed, there has been little endeavour to estimate the degree of tumour depth. Now new techniques such as ultrasonography may improve the assessment of tumour depth. While tumour length is thought to be an important indicator of cure by PDT, our multivariate analysis using factors such as the expression of bcl-2 and p53 protein, tumour length, T status, distal tumour margin, tumour location and tumour morphology did not show that tumour length is a significant indicator. We attempted multivariate analysis using the same factors except the expression of the bcl-2 and p53 protein, revealing that both T status and tumour length were significant indicators of cure by PDT (data is not shown). The expression of bcl-2 and p53 protein may be a noisy factor. Although one of the features of centrally-located early stage bronchogenic carcinoma is the difficulty of identifying the tumour (Usuda et al, 1993), an exact assessment of tumour spread is important. Light-induced fluorescence endoscope (LIFE), a new technique, may improve the assessment of the tumour spread. Further study is warranted, including endobronchial ultrasonography.

The other cause of local recurrence after PDT may be light insufficiency (Furuse et al, 1993; Sutedja et al, 1994, 1996). Indeed, the light dose in PDT protocols is more or less empirical. These illumination protocols using fixed light doses do not take into account the different concentrations of photosensitizers in the tissue concerned, as has been shown by Braichotte (1996). To raise the cure rate and reduce the local recurrence rate of PDT, it may be important to identify optimal PDT illumination.

### ACKNOWLEDGEMENTS

We are grateful to Ms Yuki Hirochi and Ms Erina Hatashita for their secretarial assistance. We also thank Mr Tomoaki Teramoto for his support in this study. This work was supported in part by a Grand-in-Aid for Cancer Research (10–28) from the Ministry of Health and Welfare, Japan.

### REFERENCES

Agarwal ML, Clay ME, Harvey EJ, Evans HH, Antunez AR and Oleinick NL (1991) Photodynamic therapy induces rapid cell death by apoptosis in L5178Y mouse lymphoma cells. *Cancer Res* **51:** 5993–5996

Agarwal ML, Larkin HE, Zaidi SIA, Mukhtar H and Oleinick NL (1993) Phospholipase activation triggers apoptosis in photosensitized mouse lymphoma cells. *Cancer Res* **53**: 5897–5902

Bennet WP, Colby TV, Travis WD, Borkowski A, Jones RT, Lane DP, Metcalf RA, Samet JM, Takeshima Y, Gu JR, Vahakangas KH, Soini Y, Paakko P, Welsh JA, Trump BF and Harris CC (1993) P53 protein accumulates frequently in early bronchial neoplasia. *Cancer Res* **53**: 4817–4822

Braichotte D, Savary JF, Glanzmann T, Monnier P, Wagnieres G and Bergh H (1996) Optimizing light dosimetry in photodynamic therapy of the bronchi by fluorescence spectroscopy. *Lasers Med Sci* **11**: 247–254

Cortese DA, Edell ES and Kinsey JH (1997) Photodynamic therapy for early stage squamous cell carcinoma of the lung. *Mayo Clin Proc* **72**: 595–602

Edell ES and Cortese DA (1992) Photodynamic therapy in the management of early superficial squamous cell carcinoma as an alternative to surgical resection. *Chest* **102**: 1319–1322

Fisher AMR, Danenberg K, Banerjee D, Bertino JR, Danenberg P and Gomer CJ (1997) Increased photosensitivity in HL60 cells expressing wild-type p53. Photochem. *Photobiol* **66**: 265–270

Furuse K, Fukuoka M, Kato H, Horai T, Kubota K, Kodama N, Kusunoki Y, Takifuji N, Okunaka T, Konaka C, Wada H and Hayata Y (1993) A prospective phase II study on photodynamic therapy with photofrin II for centrally located early-stage lung cancer. *J Clin Oncol* **11**: 1852–1857

He J, Agarwal ML, Larkin HE, Friedman LR, Xue L and Oleinick NL (1996) The induction of partial resistance to photodynamic therapy by the protooncogene bcl-2. *Photochem Photobiol* **64**: 845–852

He X, Sikes RA, Thomsen S, Chung LWK and Jacques SL (1994) Photodynamic therapy with photofrin II induces programmed cell death in carcinoma cell lines. *Photochem Photobiol* **59**: 468–473

Kato H, Konaka C, Kawate N, Shinohara H, Kinoshita K, Noguchi M, Ootomo S and Hayata Y (1986) Five-year disease-free survival of a lung cancer patient treated only photodynamic therapy. *Chest* **90**: 768–770

Kawaguchi T, Furuse K, Kawahara M, Yamamoto S and Sutedja TG (1998) Histological examination of bronchial mucosa after photodynamic therapy showing no selectivity of effect between tumor and normal mucosa. *Laser Med Sci* **13**: 265–270

Koukourakis MI, Giatromanolaki A, O'Byrne KJ, Whitehouse RM, Talbot DC, Gatter KC and Harris AL (1997) Potential role of bcl-2 as a suppressor of tumour angiogenesis in non-small-cell lung cancer. *Int J Cancer* **74**: 565–570

Luo Y and Kessel D (1997) Initiation of apoptosis versus necrosis by photodynamic therapy with chloroaluminium phthalocyanine. *Photochem Photobiol* **66**: 479–483

Mountain CF (1986) A new international staging system for lung cancer. *Chest* **89**: 225s–233s

Nishio M, Koshikawa T, Kuroishi T, Suyama M, Uchida K, et al (1996) Prognostic significance of abnormal p53 accumulation in primary, resected non-small-cell lung cancers. *J Clin Oncol* **14**: 497–502

Pass HI (1993) Photodynamic therapy in oncology: mechanisms and clinical use. *J Natl Cancer Inst* **85**: 443–456

Salgia R and Skarin AT (1998) Molecular abnormalities in lung cancer. *J Clin Oncol* **16**: 1207–1217

Separovic D, He J and Oleinick NL (1997) Ceramide generation in response to photodynamic treatment of L5178Y mouse lymphoma cells. *Cancer Res* **57**: 1717–1721

Star WM, Marijnissen H, van den Berg Blok E, Versteeg JAC, Franken KA and Reinhold HS (1986) Destruction of rat mammary tumor and normal tissue microcirculation by hematoporphyrin derivative photoradiation observed in vivo in sandwich observation chamber. *Cancer Res* **46**: 2532–2540

© 2000 Cancer Research Campaign

Sutedja G, Lam S, LeRiche JC and Postmus PE (1994) Response and pattern of failure after photodynamic therapy for intraluminal stage I lung cancer. *J Bronchol* **1**: 295–298
Sutedja G and Postmus PE (1996) Photodynamic therapy in lung cancer. A review. *Photochem Photobiol* **36**: 199–204
Usuda K, Saito Y, Nagamoto N, Sato M, Sagawa M, Kanma K, Takahashi S, Endo C and Fujimura S (1993) Relation between bronchoscopic findings and tumor size of roentgenographically occult bronchogenic squamous cell carcinoma. *J Thorac Cardiovasc Surg* **106**: 1098–1103
Walker C, Robertson L, Myskow M and Dixon G (1995) Expression of the bcl-2 protein in normal and dysplastic bronchial epithelium and in lung carcinomas. *Br J Cancer* **72**: 164–169

*© 2000 Cancer Research Campaign* 
